# Hyoscine Hydrobromate as a Hypnotic

**Published:** 1889-11

**Authors:** E. B. Potter

**Affiliations:** Physician to Monroe County Insane Asylum, Rochester, N. Y.


### Southern Medical Record.

A MONTHLY JOURNAL OF PRACTICAL MEDICINE.

☞ All communications must be addressed to R. C. WORD, Managing Editor.

"*Quicquid Præcipies Esto Brevis.*"

VOL. XIX ATLANTA, GA., NOVEMBER, 1889. NO. 11

### ORIGINAL AND SELECTED ARTICLES.

### HYOSCINE HYDROBROMATE AS A HYPNOTIC.

BY E. B. POTTER, M. D.

*Physician to Monroe County Insane Asylum, Rochester, N. Y.*

The administration of hydrobromate of hyoscine and its action as a hypnotic, has been suggested to me as a subject that would be of interest and might show where its use is indicated in the sleeplessness and delirium of some acute diseases.

Hyoscyamus was always a favorite anodyne with me when in general practice. There are always certain people on whom the effect of opium, chloral, alcohol, etc., seems to increase the nervous, irritable or sleepless conditions from which they suffer, and in such persons a full dose of fluid extract of hyoscyamus generally proved a successful remedy. Hyoscine being freely soluble makes an easy drug to use hypodermically, which is almost a necessity in prescribing for a maniacal or delirious person. Morphine is just as easily administered hypodermically, but among asylum patients, when used merely as a hypnotic, unless given in larger doses than is pleasant, on account of the functional disturbances which it causes, seems to stimulate instead of produceing rest. Chloral is a good hypnotic, but not easy of administration in many cases. Sulfonal has the same objection among patients who will not swallow what you give them. I might go through the pharmaceutical list and show that hyoscine is the easiest to administer and the surest to produce the effect sought, which is generally to get your patient to sleep. Hyoscine will produce sleep and will do it without very unpleasant sequences. Small doses seem to excite cerebral action, making a person more active and talkative. It causes dryness of the mouth and throat, and the pupils dilate to the same extent as in the use of atropia. It is only in large doses that it is hypnotic. In such doses the pulse-rate increases very fast, running up thirty, forty or fifty beats in from five to ten minutes. The patient may be more disturbed for a short time; occasionally violently so. The speech begins to thicken, the limbs become temporarily paralyzed, and in from ten to twenty minutes the patient drops into a profound sleep, which lasts from four to eight hours, and a sleep so profound as to make a family uneasy, and might worry a physician if experience had not shown that he would awake from it, probably inproved and with only the trouble of vision caused by the dilatation, and a feeling of lassitude to complain of. It does not constipate the bowels, and, if anything, increases the flow of urine. I have known of only one person who was nauseated by its use, but she would vomit after the administration of any anodyne. It does not seem to disturb the appetite, and there seems no disposition to form a habit. Most of the maniacal and sleepless patients improve in a few days so they sleep without an anodyne, and when it is dropped do not seem to miss it. It was given to two noisy and destructive patients each night for three months. They then began to improve, and its use was discontinued. One has been helping in the kitchen, and the other went home recovered, a year ago. It could be used frequently where opium and chloral are, without the danger of forming a habit. The tablet is convenient to carry, and can be used by the mouth or hypodermically, as you wish. I began using $\frac{1}{100}$ grain sulphate of hyoscyamia tablets, and increased one at a time up to five, going from $\frac{1}{100}$ to $\frac{1}{20}$ grain, and finding each increase a safe prescription and an increase in the value of the remedy. I now use hydrobromate of hyoscine, and make a solution, nine minims of which contain $\frac{1}{20}$ grain, and that is the dose I generally use, except with a violent patient who requires more to quiet a maniacal candition. It may be given in $\frac{1}{15}$ grain doses. I have given $\frac{1}{15}$ grain, and repeated it in two hours. It is a safe remedy to use. My use of it has been, of course, mostly to produce sleep in violent, destructive, or noisy patients, and those who do not sleep because of the imaginary troubles of melancholia. I have used it in three cases of chronic alcoholism where the patients had the hallucinations of sight, the great fear of harm coming to them, the sleeplessness, etc., that such persons suffer from. In two cases the patient slept and commenced to improve from the first. The other passed a quiet night from $\frac{1}{20}$ grain and slept each night after. I had to give it only three or four nights, the patient becoming quiet, sleeping, eating and getting well in a few weeks so as to go home. One case of mania, recently admitted, badly reduced by hard work and worry, was very maniacal. Her husband was sick, her child had died, and she became insane while caring for them and trying to earn their support at the same time. She had been struggling with three men all night and all day. On admission her temperature was 101, pulse irregular, skipping every fourth beat, slight dullness in the upper part of the lungs, some expectoration of mucus streaked with blood, fetid breath, and teeth covered with sordes. She had a hot bath, $\frac{1}{20}$ grain hyoscine, and was held in bed by three attendants until she was asleep. Her pulse ran up some thirty beats, and ceased to intermit. This was at 3 p. m.; at 9:30 p. m. she was awake and very violent. The temperature was 101½. She was given another $\frac{1}{20}$ gr. of hyoscine, and held in bed until she was asleep, and she had a quiet night. She did not eat anything but milk for several days, had hyoscine at night for four or five nights, and has slept well without it since. She eats some, but as she has the delusion of poison in her food, not heartily, does not cough any more; neither does her pulse intermit, and she is doing some hall work each day.

It acted very pleasantly on a patient suffering from cystitis. She was sick and vomiting, and I gave her morphia as she was suffering considerable pain. The next day, she said she was not passing any urine. I used the catheter and changed to hyoscine, thinking the morphia had caused the retention, but had to use the catheter the same, and after a day or two, found considerable quantities of mucus in the urine. Warm water was used to wash out the bladder, and hyoscine to allay the pain, giving a good night's sleep each night, and she recovered in a few days.

It is used in doses of from $\frac{1}{100}$ to $\frac{1}{50}$ grain in the nervous troubles where the bromides are used with good results. Patients suffering from melancholia sleep well when given $\frac{1}{25}$ grain by the mouth. It is an excellent remedy to quiet the excitement followig a series of convulsions in epileptic insanity, and will shorten the duration of an attack of recurrent mania because of the sleep secured. It will produce sleep when given hypodermically quicker than morphine, and is one of the best drugs used at asylums as a hypnotic. Acute diseases are not frequent in asylums, but from daily use for four years always getting sleep and never any unpleasant symptoms following, I should not hesitate to use the drug where sleep was needed, always remembering to give enough to put the patient to sleep.—*Buffalo Med. Jour.*

